# Post-colonoscopy Appendicitis: A Literature Review and Guidelines for Management

**DOI:** 10.7759/cureus.95565

**Published:** 2025-10-28

**Authors:** Navid Moghimi, Lars Joachim Lindberg

**Affiliations:** 1 Department of Gastrointestinal Surgery, University Hospital of Southern Denmark, Aabenraa, DNK; 2 Gastro Unit, Copenhagen University Hospital - Amager and Hvidovre, Copenhagen, DNK; 3 Department of Clinical Medicine, University of Copenhagen, Copenhagen, DNK

**Keywords:** abdominal pain, appendicitis, colonoscopy, diagnostic imaging, gastrointestinal endoscopy, iatrogenic disease, postoperative complications

## Abstract

Despite being rare, post-colonoscopy appendicitis is likely underdiagnosed due to nonspecific symptoms and lack of awareness. Currently, no clinical guideline exists to support early recognition. This review addresses that gap by synthesizing available evidence and proposing a structured management approach. Colonoscopy is a commonly performed procedure considered safe, although rare complications may occur. One such underrecognized complication is acute appendicitis following colonoscopy. This review synthesizes current literature on post-colonoscopy appendicitis, including incidence, pathophysiological mechanisms, clinical presentation, diagnostic considerations, and treatment strategies. We propose a structured clinical approach for early recognition to assist clinicians in distinguishing this rare condition from benign post-procedural symptoms. Increased awareness and timely imaging may reduce diagnostic delays and improve outcomes. A comprehensive literature search was conducted in PubMed, Embase, and Google Scholar. Studies published between 1980 and 2024 were screened. After reviewing abstracts from 74 articles, 21 were selected for full-text reviews, including 12 case reports and three review articles. The incidence of acute appendicitis following colonoscopy is estimated at 3.8 to 4.9 cases per 10,000 procedures. Most cases occur within 48 hours of the procedure and present with right lower quadrant pain, fever, and leukocytosis. Proposed mechanisms include increased intraluminal pressure, barotrauma, fecalith displacement, and direct appendiceal trauma. While surgical appendectomy is the primary treatment, conservative management has also been reported in selected cases. Acute appendicitis should be considered in the differential diagnosis of post-colonoscopy abdominal pain, especially when localized to the right lower quadrant. A structured approach may facilitate early recognition and prevent delayed management.

## Introduction and background

Colonoscopy is an essential procedure in modern gastroenterology, widely used for both screening and therapeutic purposes. Despite its generally favorable safety profile, a range of complications have been documented, including post-polypectomy bleeding (0.24%) and colonic perforation (0.06%) as the most common complications and, less frequently, post-polypectomy syndrome, splenic injury, and sedation-related cardiopulmonary events [1-4].

Acute appendicitis following colonoscopy is a very uncommon and possibly underreported complication. It was first described in medical literature in 1988 [5]. The condition remains poorly understood due to its infrequency and the absence of large-scale studies [6]. A recent systematic review by Tepelenis et al. summarized all published cases of post-colonoscopy appendicitis, consolidating data from individual reports and small case series [7]. In many instances, the clinical presentation mimics common post-procedural symptoms such as abdominal discomfort, bloating, or mild pain, leading to potential delays in diagnosis [8].

As colonoscopy becomes increasingly common worldwide, awareness of even the rare complications becomes crucial [9]. This review aims to provide a comprehensive overview of the literature on post-colonoscopy appendicitis, including incidence, proposed pathophysiological mechanisms, clinical presentation, diagnostic approaches, and current treatment strategies.

## Review

Methods

A narrative literature review was conducted to identify relevant studies on post-colonoscopy appendicitis. The databases PubMed, Embase, and Google Scholar were searched for articles published between 1980 and December 2024 using combinations of the keywords: "colonoscopy", "appendicitis", "post-colonoscopy appendicitis", and "complications". Additional references were identified through manual reference tracking of key articles.

Included publications comprised original case reports, case series, and systematic reviews describing acute appendicitis occurring within days after colonoscopy. Review articles were used to provide background and context. Data from the systematic review by Tepelenis et al. (2024) [7], which included 67 patients across 56 publications and represents the most comprehensive summary to date, were also integrated to support the descriptive analysis. Only original studies were used when extracting individual case details or assessing incidence estimates. Articles not in English or lacking sufficient clinical detail were excluded.

Results

Incidence of Post-colonoscopy Appendicitis and Other Complications

Acute appendicitis following colonoscopy is an extremely rare complication, with an estimated incidence ranging from 3.8 to 4.9 cases per 10,000 colonoscopic procedures [4,6,8]. Most cases occur within 48 hours after the procedure, although delayed presentations up to seven days have been reported [1,6]. A nationwide population-based study by Lin et al. identified 53 cases, with a median age of 55 years and a slight male predominance [4].

Post-colonoscopy appendicitis is markedly rarer compared to other recognized complications of colonoscopy, which may contribute to diagnostic uncertainty and potential delay in treatment. The overall complication rate of colonoscopy ranges from 0.1% to 0.3%, with the most reported adverse events being colonic perforation, post-polypectomy bleeding, and transient abdominal pain or bloating. A recent large-scale systematic review confirmed similar incidence rates across global populations and highlighted consistent trends in adverse events during the past two decades [10].

Perforation rates have been reported between 0.016% and 0.8%, particularly in therapeutic procedures involving polypectomy or dilation [11,12]. Post-polypectomy bleeding occurs in approximately 0.2-0.6% of procedures and is more likely in patients with coagulopathies or when larger polyps are removed [13].

Among elderly patients (>65 years), pooled rates of perforation were 0.078% and bleeding occurred in 0.235% of patients aged ≥65 years, with a notably higher rate observed in those over 80, although the exact percentage was not separately reported. This trend is supported by recent findings from Chubak et al., who reported an increased rate of serious post-colonoscopy complications specifically in the 76-85-year age group [14]. Mortality is extremely rare at approximately three per 100,000 procedures [12].

Because initial symptoms of post-colonoscopy appendicitis often resemble benign post-procedural discomfort, the condition may go underrecognized, underscoring the need for clinician awareness.

Pathophysiology

The pathophysiological mechanisms underlying acute appendicitis following colonoscopy are not fully understood due to the rarity of this complication. However, several hypotheses have been proposed based on case reports and expert opinions.

One leading theory involves intraluminal barotrauma resulting from insufflation of air or CO₂ during the procedure. During a colonoscopy, insufflation is essential to distend the colon for optimal visualization. However, insufflation leads to increased intraluminal pressure, which may extend into the appendiceal lumen. This elevated pressure can cause mechanical distension of the appendix, potentially leading to mucosal injury, compromised blood flow, and subsequent inflammation. In some cases, the increased pressure may also facilitate the obstruction of the appendiceal orifice by fecal material or lymphoid hyperplasia, initiating the cascade that leads to appendicitis.

A systematic review and recent case-based literature review have both highlighted that barotrauma from excessive insufflation is a plausible mechanism for post-colonoscopy appendicitis, emphasizing the importance of careful insufflation techniques during the procedure [7,15,16].

Another proposed mechanism is the dislodgement of a fecalith or other obstructive material into the appendiceal orifice during the passage of the colonoscope, especially in cases where difficult maneuvers or deep cecal intubation are required. This could lead to acute luminal obstruction, which is the classical initiating factor in the development of appendicitis [4,17].

Direct mechanical trauma to the appendiceal orifice or the cecal region during colonoscopy has also been considered a contributing factor, particularly when manipulation occurs near the cecum or in patients with a long or mobile appendix. The colonoscope tip may inadvertently strike or apply pressure to the appendiceal base, leading to localized mucosal injury, edema, or even micro-perforation, which in turn may initiate an inflammatory response [6]. In rare cases, trauma might disrupt the normal appendiceal drainage, contributing to stasis and subsequent bacterial overgrowth [18].

Risk Factors and Patient Characteristics

Understanding patient-related and procedural risk factors may help clinicians identify individuals at higher risk. Several studies have noted that increased age, male sex, and the presence of subclinical appendiceal inflammation may predispose patients to complications following colonoscopy. Anatomical considerations, such as a long or retrocecal appendix, may also contribute to diagnostic delay or procedural trauma. Previous abdominal or pelvic surgery may increase the risk of altered bowel anatomy, leading to challenging scope navigation and potential irritation near the ileocecal valve. Similarly, patients with underlying gastrointestinal disorders, such as inflammatory bowel disease (IBD) or chronic constipation, may exhibit increased mucosal vulnerability. Finally, excessive insufflation, especially with air rather than carbon dioxide, has been suggested as a potential aggravating factor due to increased luminal pressure [19].

Clinicians should maintain a high index of suspicion for appendicitis in patients presenting with localized right lower quadrant pain following colonoscopy, especially in those with these predisposing characteristics.

Clinical Presentation and Diagnostic Approaches

Acute appendicitis following colonoscopy often presents classical clinical features but in an atypical context, which may lead to delayed diagnosis. The most common symptom is abdominal pain localized to the right lower quadrant, often accompanied by fever, nausea, vomiting, and leukocytosis. However, in the immediate post-colonoscopy period, such symptoms may be mistakenly attributed to benign post-procedural discomfort, such as bloating, gas retention, or colonic spasm [4,7,8].

Symptom onset typically occurs within 48 hours after the procedure, although delayed presentations up to seven days have been documented [1,4,6]. Because post-colonoscopy appendicitis is rare and unexpected, clinicians may not initially include it in the differential diagnoses, particularly when the initial pain is mild or non-specific [8].

Physical examination often reveals localized tenderness over McBurney's point, with or without rebound tenderness. In some cases, abdominal guarding may be present [4,7,8].

Laboratory findings may show elevated inflammatory markers, including leukocytosis and elevated C-reactive protein (CRP), although these are nonspecific [6].

Imaging plays a central role in diagnosis. While ultrasound may be useful, particularly in younger or thinner patients, CT scanning with contrast is generally the most sensitive and specific modality. CT findings may include an enlarged appendix (>6 mm), wall thickening, periappendiceal fat stranding, or the presence of an appendicolith [1,7,20]. CT scanning is the preferred diagnostic approach if bowel perforation, post-polypectomy syndrome, or splenic injury is suspected [2,9].

Treatment Strategies

Surgical appendectomy remains the mainstay of treatment in nearly all reported cases of post-colonoscopy appendicitis, with the laparoscopic approach being most common. However, a few case reports describe successful conservative treatment with intravenous antibiotics and close clinical observation, particularly in hemodynamically stable patients without signs of perforation, abscess, or generalized peritonitis [4,7,8].

The decision between surgical and non-surgical management should be based on individual clinical presentation, laboratory and imaging findings, and patient-specific factors, such as age and comorbidities. Although no dedicated guidelines exist for post-colonoscopy appendicitis, the general treatment principles of acute appendicitis apply. Early surgical consultation is recommended in all suspected cases.

Checklist for When to Suspect Appendicitis After a Colonoscopy

**Figure 1:**
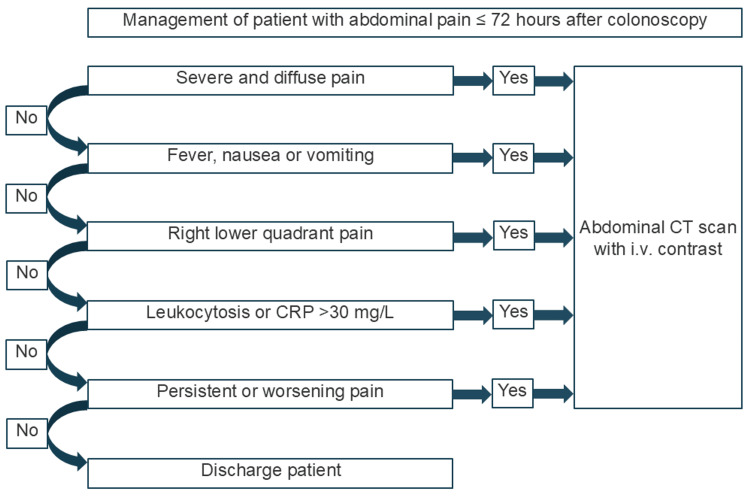
Guideline for the management of suspected post-colonoscopy appendicitis and exclusion of differential diagnosis as bowel perforation, post-polypectomy syndrome, and benign post-colonoscopic discomfort (illustration by LJL).

Although most post-colonoscopy abdominal pain is benign and self-limiting, clinicians should remain alert to more serious complications, particularly when pain localizes to the right lower quadrant and persists beyond the first several hours. The checklist and decision tool below are designed to support early recognition of post-colonoscopy appendicitis in both emergency and outpatient settings (Figure 1).

Discussion

This review underscores the need for increased clinical awareness of post-colonoscopy complications, particularly rare entities such as appendicitis, which are easily overlooked [4,7]. As the global volume of colonoscopic procedures continues to rise, clinicians must adopt a broad and systematic differential diagnostic approach when assessing post-procedural abdominal pain [1,2], and based on this review, we propose a flowchart to support systematic clinical management of post-colonoscopic symptoms.

Recent studies have emphasized the range of both minor and rare complications, such as post-colonoscopy appendicitis, underscoring the importance of maintaining clinical vigilance even in cases of seemingly benign symptoms [21,22]. Although most post-colonoscopy symptoms are benign and self-limiting, conditions like acute appendicitis may initially mimic these mild presentations, leading to diagnostic delays and potentially poorer outcomes [4,7]. Enhancing awareness, especially among gastroenterologists and emergency physicians, is therefore essential to facilitate timely recognition and appropriate management of such atypical complications [4,7,9,15,23,24].

Although post-colonoscopy appendicitis is rare, its true incidence may be underestimated due to underreporting or misattribution of symptoms to benign causes such as gas retention or transient bowel spasm [8]. Increased clinical awareness and early imaging may improve recognition and accurate diagnosis.

Proposed pathophysiological mechanisms include increased intraluminal pressure, barotrauma, fecalith displacement, and direct trauma to the appendiceal orifice [1,6,25]. These factors may lead to obstruction or inflammation of the appendix, initiating an acute process. In some instances, patients may have had preexisting subclinical inflammation of the appendix that was exacerbated or accelerated by procedural stress or mechanical factors such as pressure from scope manipulation or insufflation during colonoscopy. Overall, while these mechanisms remain speculative, it is likely that a combination of anatomical, mechanical, and predisposing inflammatory factors contributes to the development of post-colonoscopy appendicitis.

In most cases, patients present with right lower quadrant pain, fever, and leukocytosis, typically without any prior history of abdominal pathology or known risk factors. This suggests that colonoscopy itself may serve as a trigger in susceptible individuals, even in the absence of pre-existing appendiceal disease. A careful clinical history, including timing of symptom onset relative to the colonoscopy, is crucial for raising clinical suspicion.

Given the overlap between benign post-procedural symptoms and early appendicitis, we believe that the structured approach we propose can help reduce diagnostic delay. It may be particularly valuable when patients present with subtle signs in the early post-procedure period, or when initial symptoms are dismissed as expected discomfort [26].

Future research should aim to identify risk factors and procedural characteristics that may predispose patients to this complication. Greater emphasis should be placed on training and protocols that promote early detection of post-colonoscopy complications. Clinician education and increased use of diagnostic imaging when symptoms persist could significantly improve patient outcomes [8, 27]. In addition, multicenter registries with standardized reporting of rare post-endoscopic events could provide more reliable incidence data and allow meaningful analysis of risk factors across populations [28]. Prospective comparative studies are also warranted to evaluate outcomes of operative versus conservative management in this specific context, building on recent evidence from randomized appendicitis trials [29]. Furthermore, broader studies on colonoscopy-related adverse events in elderly populations may provide important insights, as age has repeatedly been associated with increased complication risk [30]. Finally, improved case-reporting standards and incorporation of such data into international endoscopy quality frameworks could help reduce underreporting and guide preventive strategies [31].

The current review is based primarily on individual case reports and small case series. As such, the findings may be subject to reporting bias, and larger prospective studies are needed to validate these observations and to validate the flowchart we propose.

In addition, collaborative international efforts to systematically document rare complications may improve recognition of patterns not visible in isolated case reports [32]. Integrating post-colonoscopy appendicitis into broader registries of iatrogenic events could help refine both preventive strategies and diagnostic algorithms [33,34]. Finally, incorporating patient-reported outcomes and real-world data may provide a more holistic understanding of the burden of this rare complication and guide evidence-based updates to colonoscopy safety guidelines [35,36].

Moreover, leveraging artificial intelligence (AI)-based tools for early symptom triage after colonoscopy may enhance timely recognition of atypical complications, including appendicitis [37]. Comparative effectiveness research focusing on diagnostic imaging modalities could further optimize cost-effective strategies for post-procedural abdominal pain [38,39]. Finally, international guideline panels should consider explicitly acknowledging post-colonoscopy appendicitis among rare but serious complications, ensuring it is included in training curricula and safety checklists [40,41,42,43].

## Conclusions

Acute appendicitis after colonoscopy is a rare but clinically relevant complication that can easily be overlooked because its symptoms often mimic benign post-procedural discomfort. Delayed recognition may increase morbidity, while early suspicion, diagnostic imaging, and prompt intervention significantly improve outcomes.

This review highlights the current evidence regarding incidence, pathophysiological mechanisms, and diagnostic challenges and introduces a structured flowchart to guide clinical decision-making. Future multicenter studies and systematic data collection are needed to better define risk factors, validate diagnostic strategies, and ensure that even rare complications are acknowledged in guidelines and training programs.
